# A Survey to Quantify the Number and Structure of Extracorporeal Membrane Oxygenation Retrieval Programs in the United States

**DOI:** 10.3390/jcm13061725

**Published:** 2024-03-17

**Authors:** Mircea R. Mihu, Laura V. Swant, Robert S. Schoaps, Caroline Johnson, Aly El Banayosy

**Affiliations:** 1 Specialty Critical Care, Advanced Cardiac Care and Acute Circulatory Support, Nazih Zuhdi Transplant Institute, Integris Baptist Medical Center, Oklahoma City, OK 73112, USA; 2 Department of Medicine, Oklahoma State University Health Science Center, Tulsa, OK 74077, USA

**Keywords:** ECMO retrieval, ECMO rescue, ECMO transport, hub and spoke model, mobile ECMO

## Abstract

(1) **Background**: Extracorporeal membrane oxygenation (ECMO) represents a potentially lifesaving support for respiratory and/or circulatory failure but its availability is limited to larger medical centers. A well-organized regional ECMO center with remote cannulation and retrieval ability can offer this intervention to patients treated at hospitals without ECMO. Information regarding the number and structure of ECMO retrieval programs in the United States is limited and there are no data regarding the size and structure of existing programs and which physician specialists perform cannulations and provide management. (2) **Methods:** We created a survey of 12 questions that was sent out to all adult US ECMO programs registered in the ELSO database. The data for the study were collected through an online survey instrument that was developed in Survey Monkey (Monkey Headquarters, Portland, OR). (3) **Results:** Approximately half of the centers that received the survey responded: 136 out of 274 (49.6%). Sixty-three centers (46%) have an ECMO retrieval program; 58 of these offer both veno-arterial (V-A) and veno-venous (V-V) ECMO, while 5 programs offer V-V ECMO rescue only. Thirty-three (52%) centers perform less than 10 ECMO retrievals per year, and only five (8%) hospitals can perform more than 50 ECMO rescues per year. Cardiothoracic surgeons perform the majority of the ECMO cannulations during retrievals in 30 programs (48%), followed by intensivists in eight (13%) programs and cardiologists in three (5%) centers. (4) **Conclusions:** Many ECMO centers offer ECMO retrievals; however, only a minority of the programs perform a large number of rescues per year. These cannulations are primarily performed by cardiothoracic surgeons.

## 1. Background

Patients who develop profound shock or severe respiratory failure refractory to mechanical ventilation may require extracorporeal life support (ECLS). Veno-arterial extracorporeal membrane oxygenation (V-A ECMO) is a rescue therapy that can stabilize patients with hemodynamic compromise, with or without respiratory failure, for days or weeks. The main indications for V-A ECMO include cardiac arrest, cardiogenic shock, post-cardiotomy shock, refractory ventricular tachycardia, support during invasive procedures, among others [1]. Veno-venous ECMO (V-V ECMO) is primarily used in cases of severe respiratory failure, such as acute respiratory distress syndrome (ARDS), where conventional mechanical ventilation is not sufficient to maintain adequate oxygenation or ventilation.

Due to its resource-intensive nature, ECMO is not widely available. ECMO requires the significant allocation of medical resources, including trained personnel, equipment, and facilities, vital to provide this specialized therapy to patients in need. ECMO is a complex and resource-intensive therapy that involves specialized equipment, highly trained personnel, and close monitoring.

Because patients’ clinical state sometimes presents an unacceptable risk for conventional transportation, specialized teams from tertiary and quaternary medical centers travel to the referring hospitals and implant ECMO in order to stabilize and then transport the patient back to the ECMO center for further care. A well-organized regional ECMO center with remote cannulation and retrieval capabilities can offer this life-saving intervention to patients treated at centers without ECLS ability. During ECMO retrievals, specialized teams from tertiary medical centers travel to referring hospitals to stabilize and place patients on ECMO. The patient is then transported back to the ECMO center for further care.

Performing ECMO cannulations at institutions without ECMO capability and the transport of patients on mechanical circulatory support adds an extra degree of complexity to the already complex task of transporting critically ill patients [2].

Per ELSO guidelines, ECMO transportation is divided into primary, secondary, tertiary, and inter-facility ECMO transfer. In primary ECMO transportation, a mobile ECMO team travels to an outside facility, cannulates and initiates ECMO and, after initial stabilization, the patient is transferred to the ECMO regional center. Secondary ECMO transportation is when a patient is already supported with ECMO but must be transferred to another facility on ECMO support, for a higher level of care. Tertiary ECMO transportation is when an institution has a patient with an ECMO indication and a rescue ECMO team from a regional ECMO center with retrieval capability travels to the facility, places the patient on ECMO, and then transports the patient to a different ECMO center due to limited ECMO capacity at their hospital [2].

Previous studies have shown that ECMO outcomes are better when patients are being cared for at specialized hospitals with a high capacity and expertise [3]. ECMO retrievals in the US have been conducted for decades, as described in the existing literature [4,5,6,7,8,9]. Information regarding the number and structure of ECMO retrieval programs in the United States is limited. In addition, there are no data regarding the size and structure of existing programs, which physician specialists perform cannulations, and which providers manage patients during the retrieval process. Furthermore, the number of ECMO rescues and types of ECMO configurations that are performed by centers with this capability are not widely published. Even less is known about the number of ECMO centers that perform ECMO retrievals. Multiple ECMO programs from the United States describe their experience in the literature; however, a report that compiles this information is not available.

To better characterize ECMO retrieval programs and regional availability in the United States, we performed a survey of adult ECMO centers affiliated with the Extracorporeal Life Support Organization (ELSO) to gain vital information on the programs’ structures, sizes, and capabilities and our findings are summarized in this report.

## 2. Materials and Methods

We conducted a cross-sectional, national survey of the ECMO centers in the United States, focusing on ECMO retrieval practices. We created a survey that was sent out to all adult ECMO programs registered in the ELSO database. The ELSO directory was used to retrieve each center’s email addresses of the ECMO program directors or ECMO program managers. The survey was assessed according to the methodology of Burns et al. [10], and 4 experts in the field of mechanical circulatory support gave feedback on the content and structure. The study was reviewed by the Institutional Review Board and deemed exempt.

The final survey contained 12 questions that were formulated to obtain information on the types of ECMO offered by each institution and whether institutions had ECMO retrieval programs. Additional information about whether the program could perform retrievals 24/7, if the respective institution offered both V-A and V-V ECMO rescue, and the number of retrievals performed per year was also attained. In addition, details about the distance traveled for retrieval, specialty of the cannulating physician, the provider in charge of the patient’s management during the transportation, size of the program, and type of institution that offered the service were requested (questionnaire included in Supplementary Materials).

The data for the study were collected through an online survey that was developed using Survey Monkey (Monkey Headquarters, Portland, OR, USA). A link to the online survey was emailed to the ECMO manager and/or ECMO program director. A reminder email containing the survey link was resent to the programs that had not completed the survey at 1, 2, and 3 months after the initial email. The survey was anonymous. The questions were designed to gather information on the number and structure of ECMO retrieval programs in the US. All responses were analyzed using Microsoft Excel 2019 MSO Version 2402 (Redmond, WA, USA) and compiled descriptively in this report.

Each center was asked to submit one response only. Responses were exported anonymously from the survey tool 1 month after the last reminder email. The number of responses to each question was recorded, and each question was analyzed separately.

## 3. Results

The survey was sent to 274 adult ECMO centers in the United States and there were 136 respondents (49.6% response rate) from 41 states. All descriptive information in this report refers only to those programs that responded to the survey. All 136 centers offer ECMO, one center offers V-A ECMO only, and two centers offer V-V ECMO only, while 133 (97.8%) centers offer both V-A and V-V ECMO. Out of the respondents, 68 (50%) centers manage less than 50 cases per year, 43 (31%) institutions manage 50 to 100 cases and 25 (19%) ECMO programs manage more than 100 cases per year. Intensivists manage the ECMO patients in 35 (26%) programs, cardiothoracic surgeons (CTSs) in 7 (5%) programs, cardiologists in 2 programs, while a multidisciplinary team including cardio-thoracic surgeons, non-CTSs, intensivists, and/or cardiologists manage ECMO patients in the rest of the centers.

Out of the total number of institutions, 63/136 centers (46%) have an ECMO retrieval program. Sixty programs offer ECMO retrieval 24 h/day while three centers provide the service during daytime hours only. In terms of retrieval types, V-A and V-V ECMO is offered by 58 (92%) centers while 5 (8%) programs offer V-V ECMO rescue only. Out of the 63 centers that offer retrieval services, 33 (52%) perform less than 10 ECMO retrievals per year, 16 (25.5%) centers perform between 10 and 30 ECMO rescues per year, 9 out of 63 (14%) centers complete 31 to 50 ECMO retrievals and only 5 hospitals are performing more than 50 ECMO retrievals per year.

Four programs (6%) retrieve patients from within a 10-mile radius, 19 (30%) centers retrieve from up to a 100-mile radius, while 40 (64%) ECMO retrieval programs can travel to and rescue patients from a distance greater than 100 miles.

CTSs perform the majority of the ECMO cannulations during retrievals in 30 programs (48%). Out of the programs in which CTSs cannulate, 19 (63%) are university hospitals. The second largest specialty service performing cannulations during ECMO rescue are intensivists, performing cannulations in eight (13%) programs out of the respondents. This is followed by cardiologists in three programs (5%) and non-CTSs in one program. A combination of CTSs, intensivists, cardiologists, and non-CTSs perform cannulations in the remainder (32.5%) of the centers that offer retrieval. The cannulating physician is responsible for the management of the ECMO patient during transport back to the institution in 52 of the 63 programs (82.5%), while in 11 (17.5%) cases, another specialist (perfusionist, intensivist, ECMO nurse practitioner, etc.) is responsible for the management of the patient during transport.

Out of the centers with ECMO retrieval programs, 20 (32%) hospitals are large-volume ECMO centers that manage more than 100 ECMO cases per year, 27 (43%) are medium-sized programs, running 50 to 100 ECMO cases per year, and 16 (25%) hospitals manage less than 50 ECMO patients per year. Out of the centers that offer ECMO retrievals, 49% are university hospitals, 36.5% are private institutions, while 14.5% are university-affiliated or community hospitals.

## 4. Discussion

ECMO is performed most successfully in high-volume ECMO centers for best resource use, safety, effectiveness, and outcomes [11,12]. ECMO referral centers should be organized in a hub and spoke model, as recommended by experts in the field [12].

The formation of advanced cardiac care systems that offer all aspects of cardiac care, including short-term mechanical circulatory support (ECMO, percutaneous ventricular assist devices), durable ventricular assist devices, and transplants, is essential and may affect the outcomes of patients with profound cardiac failure [1]. Moreover, for patients suffering from acute respiratory failure, Peek et al. described a significant improvement in survival of patients transferred to a specialized center for consideration of ECMO intervention compared with continued conventional ventilation [3].

Traditionally, the large ECMO centers are university hospitals. Interestingly, in our survey, the large ECMO centers were equally divided between private institutions and university hospitals.

Per the current ELSO guidelines for the transport and retrieval of ECMO adult patients, the composition of the retrieval team should include a team leader, cannulating provider (can be the leader), ECMO specialist, and a medical transport team/emergency medical service [2].

Many programs in the US provide ECMO services but less than half have a retrieval program. As this survey indicated, the majority of programs can perform both V-A and V-V ECMO retrieval, and are available 24 h a day. However, most centers perform only a small number of retrievals, with more than half of the programs completing <10 rescues per year. As previously shown in the literature, high-volume adult ECMO centers have demonstrated improved rates of survival compared to centers performing fewer cannulations [5,11,12].

The ECMO regional centers have an obligation to perform outreach, education, and ensure accessibility to ECMO services. This represents the foundation of a successful ECMO retrieval program. Furthermore, the large ECMO hubs have a responsibility to ensure the safe and effective delivery of ECMO therapy, and to provide high-quality ECMO care in order to improve patient outcomes. These obligations are critical for maintaining the effectiveness and sustainability of ECMO programs and meeting the needs of critically ill patients.

Traditionally and to date, ECMO cannulations are performed by CTSs; however, the increased utilization of ECMO [13] and limited availability of CTSs, especially during the COVID-19 pandemic, have created a mismatch between the demand for ECMO and accessibility of CTSs for remote cannulations, necessitating the involvement of other services to perform cannulations and retrievals. As previously shown in the literature, cannulations can be performed safely by other non-surgical specialties [9,14,15,16,17,18]. Based on this review, the number of specialists that have acquired this skillset appears to be increasing. Retrieval programs have grown in size and in number and have become more interdisciplinary, which is essential to meet the growing demand for ECMO in the US, especially in the context of the future estimated shortage of CTSs by 2035 and projected growing number of caseloads for each CTS by 121% [19].

As previously described in the literature [20], given the nature of this survey-based study, a limitation includes its inability to describe the programs to which the survey was distributed. Survey questions were left to the respondent for interpretation; however, the structure of the questions was created to limit bias, and were both open-ended and multiple choice, to gather consistent information from each respondent. Since about 50% of the programs responded to the survey across 41 states, the data extracted from the respondents are expected to be representative of most ECMO programs in the US, and associated with results that are more generalizable.

## 5. Conclusions

To the best of our knowledge, this is the first publication characterizing ECMO retrieval programs in the US. Many ECMO centers offer ECMO retrievals; however, only a minority of programs perform large numbers of rescues per year and CT surgeons perform most of the ECMO cannulations. In the future, creating a network of hospitals that provide retrievals may be of benefit to learn and improve from each program’s successes, failures, and new innovations. This may in turn create a unified ECMO network to best serve patients in preparation for a rise in demand related to increasing heart disease and potential pandemics.

## Data Availability

Data available on request due to restrictions eg privacy. The data presented in this study are available on request from the corresponding author. The data are not publicly available. The survey was anonymous.

## Supplementary Materials

The following supporting information can be downloaded at https://www.mdpi.com/article/10.3390/jcm13061725/s1.

## References

[1] Guglin M., Zucker M.J., Bazan V.M., Bozkurt B., El Banayosy A., Estep J.D., Pinney S.P. (2019). Venoarterial ECMO for Adults: JACC Scientific Expert Panel. J. Am. Coll. Cardiol..

[2] Labib A., August E., Agerstrand C., Frenckner B., Laufenberg D., Lavandosky G., Fajardo C., Gluck J.A., Brodie D. (2022). Extracorporeal Life Support Organization Guideline for Transport and Retrieval of Adult and Pediatric Patients with ECMO Support. ASAIO J..

[3] Peek G.J., Mugford M., Tiruvoipati R., Wilson A., Allen E., Thalanany M.M., Elbourne D. (2009). Efficacy and economic assessment of conventional ventilatory support versus extracorporeal membrane oxygenation for severe adult respiratory failure (CESAR): A multicentre randomised controlled trial. Lancet.

[4] Biscotti M., Agerstrand C., Abrams D., Ginsburg M., Sonett J., Mongero L., Takayama H., Brodie D., Bacchetta M. (2015). One Hundred Transports on Extracorporeal Support to an Extracorporeal Membrane Oxygenation Center. Ann. Thorac. Surg..

[5] Bonadonna D., Barac Y.D., Ranney D.N., Rackley C.R., Mumma K., Schroder J.N., Daneshmand M.A. (2019). Interhospital ECMO Transport: Regional Focus. Semin. Thorac. Cardiovasc. Surg..

[6] Bryner B., Cooley E., Copenhaver W., Brierley K., Teman N., Landis D., Rycus P., Hemmila M., Napolitano L.M., Haft J. (2014). Two decades’ experience with interfacility transport on extracorporeal membrane oxygenation. Ann. Thorac. Surg..

[7] Dalia A.A., Axtel A., Villavicencio M., D’Allesandro D., Shelton K., Cudemus G., Ortoleva J. (2019). A 266 Patient Experience of a Quaternary Care Referral Center for Extracorporeal Membrane Oxygenation with Assessment of Outcomes for Transferred Versus In-House Patients. J. Cardiothorac. Vasc. Anesth..

[8] Foley D.S., Pranikoff T., Younger J.G., Swaniker F., Hemmila M.R., Remenapp R.A., Copenhaver W., Landis D., Hirschl R.B., Bartlett R.H. (2002). A review of 100 patients transported on extracorporeal life support. ASAIO J..

[9] Mihu M.R., Maybauer M.O., Cain K., Swant L.V., Harper M.D., Schoaps R.S., Brewer J.M., Sharif A., Benson C., El Banayosy A.M. (2023). Bridging the gap: Safety and outcomes of intensivist-led ECMO retrievals. Front. Med..

[10] Burns K.E., Duffett M., Kho M.E., Meade M.O., Adhikari N.K., Sinuff T., Cook D.J., for the ACCADEMY Group (2008). A guide for the design and conduct of self-administered surveys of clinicians. Can. Med. Assoc. J..

[11] Barbaro R.P., Odetola F.O., Kidwell K.M., Paden M.L., Bartlett R.H., Davis M.M., Annich G.M. (2015). Association of hospital-level volume of extracorporeal membrane oxygenation cases and mortality. analysis of the extracorporeal life support organization registry. Am. J. Respir. Crit. Care Med..

[12] Combes A., Brodie D., Bartlett R., Brochard L., Brower R., Conrad S., De Backer D., Fan E., Ferguson N., Fortenberry J. (2014). Position paper for the organization of extracorporeal membrane oxygenation programs for acute respiratory failure in adult patients. Am. J. Respir. Crit. Care Med..

[13] van Diepen S., Katz J.N., Albert N.M., Henry T.D., Jacobs A.K., Kapur N.K., Kilic A., Menon V., Ohman E.M., Sweitzer N.K. (2017). Contemporary Management of Cardiogenic Shock: A Scientific Statement from the American Heart Association. Circulation.

[14] Burrell A.J.C., Pilcher D.V., Pellegrino V.A., Bernard S.A. (2018). Retrieval of Adult Patients on Extracorporeal Membrane Oxygenation by an Intensive Care Physician Model. Artif. Organs.

[15] Liu N., Han X., Huang R., Yu C., Fang M., Yang W., Zha Y., Shao M. (2023). Intensivist-Led Transportation of Patients on Extracorporeal Membrane Oxygenation: A Single Center Experience. ASAIO J..

[16] Kraai E., Teixeira J.P., Patel I.A., Wray T.C., Mitchell J.A., George N., Kamm A., Henson J., Mirrhakimov A., Guliani S. (2022). An Intensivist-Led Extracorporeal Membrane Oxygenation Program: Design, Implementation, and Outcomes of the First Five Years. ASAIO J..

[17] Conrad S.A., Grier L.R., Scott L.K., Green R., Jordan M. (2015). Percutaneous cannulation for extracorporeal membrane oxygenation by intensivists: A retrospective single-institution case series. Crit. Care Med..

[18] Kouch M., Green A., Damuth E., Noel C., Bartock J., Rosenbloom M., Schorr C.D., Rios R.R., Loperfido N.B., Puri N. (2022). Rapid Development and Deployment of an Intensivist-Led Venovenous Extracorporeal Membrane Oxygenation Cannulation Program. Crit. Care Med..

[19] Moffatt-Bruce S., Crestanello J., Way D.P., Williams T.E. (2018). Providing cardiothoracic services in 2035: Signs of trouble ahead. J. Thorac. Cardiovasc. Surg..

[20] van Minnen O., Jolink F.E., van den Bergh W.M., Droogh J.M., Lansink-Hartgring A.O., Dutch ECLS Study Group (2023). International Survey on Mechanical Ventilation During Extracorporeal Membrane Oxygenation. ASAIO J..

