# Supplementary material for: A Survey to Quantify the Number and Structure of Extracorporeal Membrane Oxygenation Retrieval Programs in the United States
Source: J Clin Med. 2024 Mar 17;13(6):1725. doi: 10.3390/jcm13061725 (PMC10971712; doi:10.3390/jcm13061725)
Supplement: Supplementary file 1 [file jcm-13-01725-s001.zip › jcm-2880705-supplementary.pdf]

1. Does your program offer ECMO?
   Yes
   No

2. Type of ECMO offered

   V-V ECMO

   V-A ECMO

3. Do you have an ECMO retrieval program, where you cannulate patients at outside hospitals and transport them to your institution?
   Yes
   No

4. Do you offer ECMO retrievals 24/7?
   Yes
   No

5. Do you offer both V-V and V-A ECMO retrievals?
   Yes
   No

6. How many retrievals do you average per year?

   <10
   10-30
   31-50
   >50

7. What is the maximum distance that you travel to retrieve patients?

   <10 miles
   10-100 miles
   >100 miles

8. Who performs remote cannulations during retrievals. (you may choose more than one answer)

   Cardiothoracic surgeon
   Non-CT surgeon
   Intensivist
   Cardiologist

9. Is the cannulating physician also responsible for management prior to and during transport? If not, who manages the patient prior to arrival to your institution?

10. Who is responsible for patient management while on ECMO at your institution. (You may choose more than one answer)

Cardiothoracic surgeon
Non-CT surgeon
Intensivist
Cardiologist

11. What is the size of your ECMO program?

Low volume (<50)
Medium size (51-100)
High volume (> 100)

12. How would you describe the institution that houses your ECMO program ?

University hospital
University affiliated, community hospital
Private institution
Veteran Administration
